# Use of #NutritionFacts to promote evidence-based nutrition information: X (formerly Twitter) hashtag analysis study

**DOI:** 10.3389/fpubh.2023.1255706

**Published:** 2023-12-06

**Authors:** Maima Matin, Tanuj Joshi, Michael Greger, Farhan Bin Matin, Artur Jóźwik, Agnieszka Wierzbicka, Jarosław Olav Horbańczuk, Harald Willschke, Atanas G. Atanasov

**Affiliations:** ^1^Institute of Genetics and Animal Biotechnology of the Polish Academy of Sciences, Magdalenka, Poland; ^2^Department of Pharmaceutical Sciences, Kumaun University, Nainital, Uttarakhand, India; ^3^NutritionFacts.org, Takoma Park, MD, United States; ^4^Department of Pharmacy, East West University, Dhaka, Bangladesh; ^5^Department of Technique and Food Product Development, Warsaw University of Life Sciences (WULS-SGGW), Warsaw, Poland; ^6^Ludwig Boltzmann Institute Digital Health and Patient Safety, Medical University of Vienna, Vienna, Austria; ^7^Department of Anaesthesia, Intensive Care Medicine and Pain Medicine, Medical University Vienna, Vienna, Austria

**Keywords:** nutrition, X (Twitter), health, social media, public health, hashtags

## Abstract

Nutrition is a key determinant of health, and the dissemination of reliable nutrition information to consumers is of great importance for public health. Especially with the rise of digital communication technologies and the wide-spread online misinformation, the provision of qualitative science-based information related to diet is of great importance. The NutritionFacts.org has been established as a prominent online source of evidence-based nutrition information. In this work we aimed to investigate the use of the associated hashtag #NutritionFacts on X (formerly Twitter) over a 5 years period, from 10th of April 2018 to 10th of April 2023. The conducted analysis with the use of Symplur Signals revealed that 18,998 tweets mentioning #NutritionFacts were posted by 6,136 X users, generating a total of 50,348,223 impressions (views). Both institutional and individual accounts were broadly participating in the dissemination of #NutritionFacts tweets, and the user location profiling indicated wide international engagement with the hashtag. This work indicates that #NutritionFacts has been established as an important hashtag utilized on X for the dissemination of evidence-based information related to nutrition.

## Introduction

Nutrition plays a vital role in maintaining health (1, 2). A balanced or nutritional diet is one which provides all the important macro and micro nutrients which are essential for the normal functioning of the human body on a daily basis (1). The essential components of a balanced diet are: carbohydrates, proteins, vitamins, fats, minerals and fibers (3). Poor eating habits lead to deteriorated health. Ignoring healthy food choices and consuming highly processed foods and drinks high in fats and sugars can lead to nutritional deficiencies (2). The concept of balanced diet postulates that optimum health can be achieved by eating a variety of qualitative foods rich in various nutrients (3). People in today’s world have better health facilities than previous generations yet they suffer from various health complications that are due to poor dietary habits (4, 5).

Scientific evidence is very important for policy making and the establishment of effective practices associated with health care. The same principle is also applicable to nutrition. However, in the digital age general public faces challenges to keep being well-informed on diverse health-related topics based on qualitative scientific information. Nowadays anyone with access to social media and basic recording equipment can become communicator on health-related claims that can have or not have support from scientific evidence. Thus, information received by the audiences is often misrepresented and false in nature (6). On this background, the communication of evidence-based nutrition information assumes a highly significant role with respect to nutrition and health. The evidence-based framework with respect to nutrition comprises of the following key components: Nutrient intake recommendations and dietary guidelines, clinical practice guidelines, food standards and health claims and systemic reviews. There are many challenges with respect to the above mentioned components of evidence-based nutrition. Thus, up-gradation of the above mentioned components of evidence based nutrition is very necessary (6–11).

There are many websites on the internet that aim to inform the population regarding nutrition science. One fine example of such a website is “Nutritionfacts.org.” Nutritionfacts.org is a nonprofit and scientific organization started by the renowned American physician Dr. Michael Greger (disclosure: one of the authors of this work). This organization spreads current research on nutrition and healthy eating through podcasts, blogs, videos and infographics. It also communicates information on healthy eating, longevity and disease prevention through nutrition on social networking platforms. Nutritionfacts.org was started with the help of seed money from Jesse & Julie Rasch Foundation. At present the website thrives on the money from individual donors (12). This website emphasizes the fact that instead of consuming expensive drugs or undergoing surgical procedures for treatment of diseases, many medical conditions could be counteracted by applying appropriate nutrition plans and maintaining a healthy lifestyle. Along this line, the aim is to keep the general public informed with up-to-date information on nutrition by presenting peer-reviewed studies-derived facts and research on nutrition in a simple manner so that a layman can also understand concepts related to nutrition with relation to health and disease (12).

X (formerly Twitter) is a social networking platform that is very popular throughout the world. It is used for sharing of information and opinions on countless topics in the form of short comments (tweets). Through X people can share their thoughts in the form of micro blogs and this is a very efficient way to spread information. X communications can have powerful effects on shaping public opinions (13). There are many topics trending on X in any moment, and to allow people to easily discover particular topics of interest hashtags are often used for labeling relevant posts. Hashtags represent words or phrases (written without intervals) preceded by a hash sign “#.” Hashtags help in categorizing and grouping thematically-related posts, and represent a very helpful tool for searching tweets on a topic of one’s interest trending on X (13, 14). The hashtag #NutritionFacts in particular has been established for labeling of content related to evidence-based nutrition. Health-related information nowadays is circulated in all social networking platforms. X in particular represents one of the highly popular platforms for sharing information regarding nutrition and health (15). From one side, X is a good forum where the health practitioners, doctors, researchers, patients and the general public can interact with each other and exchange qualitative information. From the other side, information spread on X can also be false, misleading, or incomplete. Thus, health-concerned users should thoroughly screen the information available on X for accuracy. X can become a very useful platform for providing information regarding nutrition and can change the lives of many people if the information spread through it is reliable (16, 17). Importantly, X (formerly Twitter) bears a great potential both as a channel of communication with the audience and as a mean of raising awareness of many social and health-related issues. Although its effectiveness has been shown to be limited (18), and recent ownership-change presented some new challenges to science-communicators (19), this platform still has a clear platform to reach citizens and change their health-related behavior (20, 21), which makes it a suitable avenue for studies investigating the use of hashtags promoting science-based information related to health.

Social media hashtag analysis in general has been established as a valuable tool to monitor online discussions focused on a specific topic (22, 23), as well as an instrument to evaluate the effectiveness of interventions aiming to popularize specific topics (24–26) or to aid the creation of new online communities (27, 28). Several hashtag analyses works with relevance to food and eating previously explored the use of #HealthyLifestyle on TikTok (29), the use of five popular hashtags related to nutrition and dietetics (#nutrition, #nutritionist, #instadiet, #diet, and #dietitian) on Instagram (30), and the use of hashtags related to eating disorder awareness campaigns (#wakeupweightwatchers, #eatingdisorderawarenessweek, #eatingdisorderawareness, #EDAW, and #eatingdisorder) on X (formerly Twitter) (31). In the area of evidence-based nutrition in particular, previous work focused on characterization of #heartfailure-mentioning tweets and the attitude of physicians and registered dietitian nutritionists toward the use of social media for dissemination of evidence-based nutrition practice guideline with relevance to heart failure patients (32). However, the use of #NutritionFacts has not been studied up to now on any social media platform.

Overall, the spread of scientifically-correct nutrition information on social networking sites like X is of great importance, since such information has great implications for public health (6, 16, 17). On this background, we hypothesized that analysis of the #NutritionFacts tweets shared over a five year period will yield new insights on the patterns of use of this hashtag as a tool to promote evidence-based nutrition information on X, and thereby aimed to obtain quantitative data on number of shared tweets and posting users, number of generated impressions, stakeholder segmentation and location of the participating accounts, most frequently hyperlinked external web domains, most frequently used terms and co-occurring hashtags, features (likes, re-shares, comments) of the most engaging posts, and characteristics of the top influencer-accounts of the #NutritionFacts tweets.

## Methods

### Hashtag analysis

The target time-period covered five years, from 10th of April 2018 to 10th of April 2023. Upon registration of the hashtag #NutritionFacts with Symplur Healthcare Hashtag Project, the aim was that it would be used for the promotion of nutrition and health-related communication on X (33). The analysis of tweets shared in the study period was performed with Symplur Signals, providing user interface accessible online, and representing a well-established hashtag analysis tool enabling long-term recording and monitoring of tweets mentioning specific hashtags registered with the Healthcare Hashtag Project (33–35). The conducted analysis performed with the standard settings of Symplur Signals involved determination of the total number of shared tweets, posting accounts (X users), and impressions (i.e., views of tweets). Trending terms and co-occurring hashtags, identity and features (likes, re-shares, comments, presence of image or video clip) of the most engaging posts, characteristics of the top influencer-accounts, most frequently hyperlinked external web domains, as well as geolocations of the posting accounts and their segregation into healthcare stakeholder groups was also determined with Symplur Signals. All tweets mentioning #NutritionFacts were included in the analysis without application of filters posing any restrictions on language, location, or other parameters. Tables and figures were drafted and optimized with MS Excel and Adobe Photoshop, respectively.

### Ethical aspects

The present work is exempted from ethics review since it just examines pre-existing publicly available data and did not involve any prospective collection of data from human participants. All data presented in this work are anonymized and no information related to specific X user accounts is revealed.

## Results

**Figure 1:**
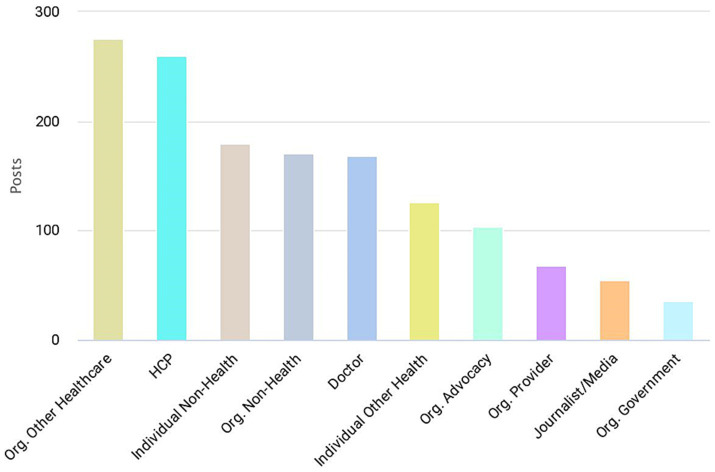
Number of tweets shared by the top ten healthcare stakeholders. Definitions according to the Symplur glossary: *Org. Other Healthcare*: organizations fulfilling roles within the healthcare industry but not providing direct clinical care; *HCP*: those believed to be other healthcare professionals (i.e., nurses, dietitians, respiratory therapists, nurses, pharmacists, etc.); *Individual Non-Health*: Person not known to be directly working in the healthcare industry; *Org. Non-Health*: All organizations not falling into an established category; *Doctor*: Those believed to be licensed, MDs, DOs, PhDs who bill directly for services. Also includes medical residents; *Individual Other Health*: Person working in the healthcare industry in a nonclinical role; *Org. Advocacy*: an organization focused on a specific set of health issues or medical specialty for the purpose of support, guidance, and education; *Org. Provider*: Inpatient facilities, medical groups, labs, imaging centers, and other outpatient facilities; *Journalist/Media*: Person whose profession is journalism or other news-related media. Doctors who are editors of journals do not get this label; *Org. Government*: government accounts at local, state, and national levels.

The performed analysis revealed that 18,998 tweets mentioning #NutritionFacts were posted by 6,136 X users for the 5 years study-period (between 10th of April 2018 and 10th of April 2023). These tweets generated a total of 50,348,223 impressions (views). Healthcare stakeholders analysis revealed that the top ten list (by number of shared tweets; Figure 1) contained multiple organizational as well as individual user account categories, with the three leading categories being organizations fulfilling roles within the healthcare industry but not providing direct clinical care (*Org. Other Healthcare*; 276 tweets posted in the study period), healthcare professionals (*HCP*; 260 tweets), and persons not known to be directly working in the healthcare industry (*Individual Non-Health*; 180 tweets).

From the users who posted #NutritionFacts-containing tweets in the study period and revealed location-information in their X-profiles (Table 1), most were located in The United States of America (1,175 users), followed by India (448), Canada (139), Nigeria (47), and South Africa (30).

**Table 1:** Locations of users that posted tweets mentioning #NutritionFacts in the study period.

No.	Country	Users
1	The United States of America	1,175
2	India	448
3	Canada	139
4	Nigeria	47
5	South Africa	30

The top 10 most commonly co-occurring hashtags in the #NutritionFacts-containing tweets include #nutrition, #nutritiontips, #health, #healthyfood, #Nutritionist, #healthy, #healthylifestyle, #HealthyEating, #nutritionplan, and #food (Table 2).

**Table 2:** Top co-occurring hashtags of the tweets mentioning #NutritionFacts in the study period.

No.	Hashtag	Occurrences	No.	Hashtag	Occurrences
1	#nutrition	4,180	6	#healthy	1,230
2	#nutritiontips	2,052	7	#healthylifestyle	1,076
3	#health	1,829	8	#HealthyEating	1,040
4	#healthyfood	1,608	9	#nutritionplan	989
5	#nutritionist	1,513	10	#food	826

**Figure 2:**
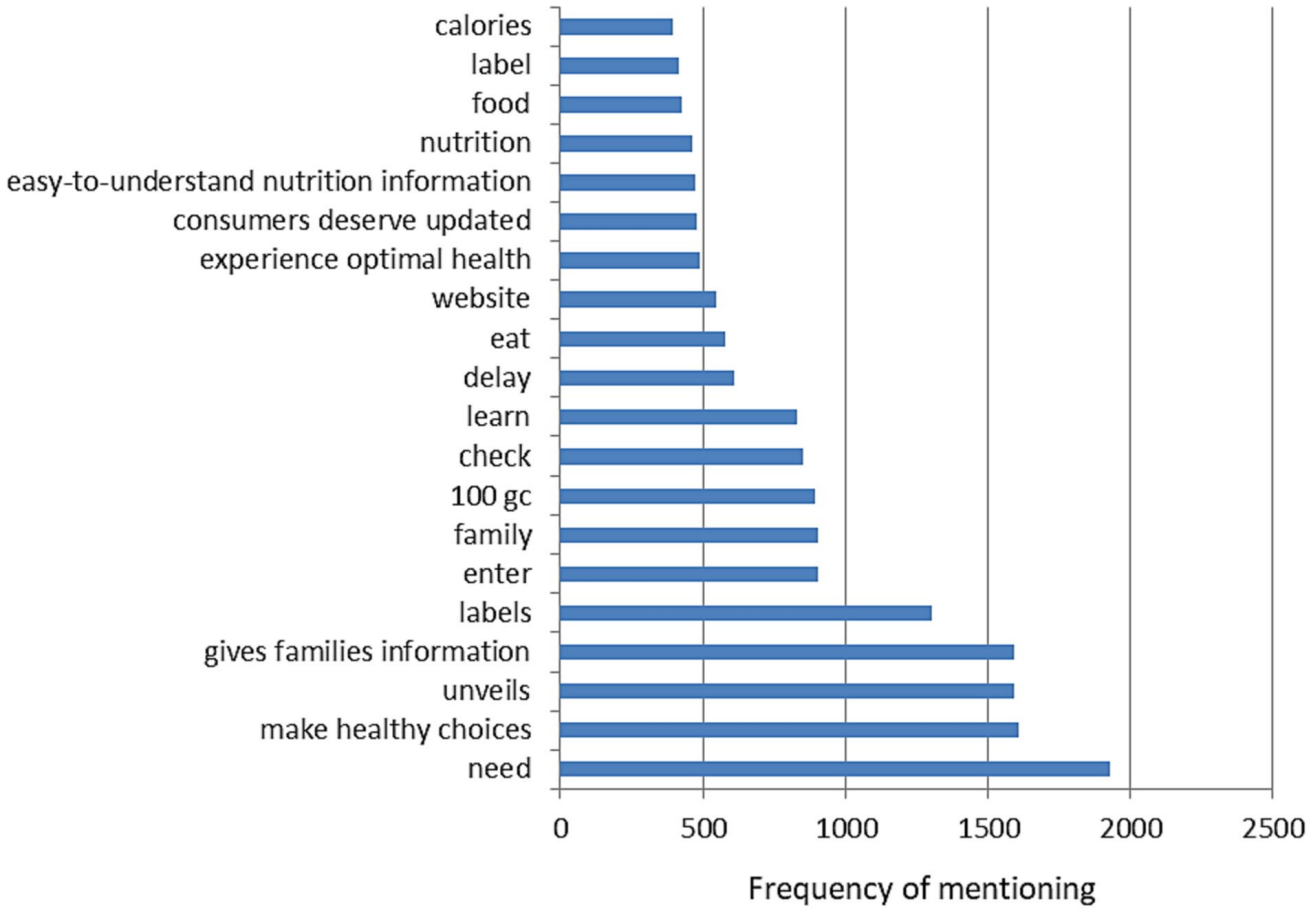
Top 20 trending terms of the #NutritionFacts tweets.

Analysis of the top 20 trending terms (Figure 2) in the #NutritionFacts-containing tweets that were shared in the study period indicated as most frequently shared the terms “need” (1927 occurrences), “make healthy choices” (1605), “unveils” (1592), “gives families information” (1589), “labels” (1301), “enter” (903), “family” (900), “100 gc” (889), “check” (851), “learn” (829), “delay” (606), “eat” (575), “website” (542), “experience optimal health” (487), “consumers deserve updated” (473), “easy-to-understand nutrition information” (468), “nutrition” (461), “food” (424), “label” (412), and “calories” (391).

Further analysis of external web domains most frequently hyperlinked in the #NutritionFacts-containing tweets revealed that the top five were instagram.com (555 shares), youtube.com (210 shares), nutritionfacts.org (135 shares), fda.gov (110 shares) and checkyourfood.com (92 shares). The most engaging tweet (64 re-shares, 105 likes, and 6 comments) was featuring a meme-style image ironically presenting features of Trump supporters in a way resembling “Nutrition facts” on a food label, with co-occurring hashtags #TrumpUblican and #Unhealthy. While the latter tweet also featured the most shared image, the most shared video clip (20 shares) was entirely focused on a nutrition-related topic and was promoting health benefits of tamarind. The most shared hyperlink (posted 65 times) in the #NutritionFacts tweets was to a fda.gov website article entitled “The Nutrition Facts Label / What’s in it for you?,” while the tweet that received most comments (16 comments) was featuring an image encouraging the viewers to identify nutrition-related words in a visual puzzle.

The top 10 influencer-accounts (by number of generated views/impressions) generated from a total of 13,585,060 views (rank 1, overall 16 posted #NutritionFacts tweets) to a total of 648,262 views (rank 10, overall 7 posted #NutritionFacts tweets). Within these top 10 influencer accounts just 3 were personal, while the other 7 belonged to organizations such as Mayo Clinic, The United States Food and Drug Administration, and The Academy of Nutrition and Dietetics, among others. Interestingly, the account of the nutritionfacts.org founder Dr. Michael Greger was not ranked in the top 10 (it ranked fifteenth, with a total of 414,859 generated views).

## Discussion

We analyzed the X posts mentioning the hashtag #NutritionFacts in a 5 years period, from 10^th^ of April 2018 to 10^th^ of April 2023. There were 18,998 #NutritionFacts-mentioning tweets that were shared in the study period by 6,136 X users, yielding 50,348,223 impressions (views). This is the first work that examines X activity associated with the use of #NutritionFacts. Previous diet-related X analysis have examined the nutrition-information relevance of 298 tweets containing the hashtag #heartfailure (32) 2,886 tweets of dietitians and users sharing nutrition information during COVID-19 (36) and 81,249 tweets that contained combination of the terms ‘food’ and ‘poverty’ during the COVID-19 pandemic (37). Interestingly, the healthcare stakeholders analysis performed by us (Figure 1) indicated that the top ten list contained diverse organizational categories with the most prominent stakeholder being organizations fulfilling roles within the healthcare industry but not providing direct clinical care (*Org. Other Healthcare*). Thus, it is apparent that information associated with the #NutritionFacts tweets is notably advocated at organizational level. For comparison, doctors were previously found to be the leading stakeholder sharing tweets mentioning #SaludTues (hashtag advocating for Latinx health equity) (38) and #PsychTwitter (hashtag aimed for the dissemination of psychiatric knowledge and information) (34).

Concerning the locations of the users who shared #NutritionFacts-containing tweets in the study period (Table 1), not surprising in the top three were countries with big share of English-speaking population (The United States of America, India, and Canada), while it is also interesting to note the presence of users from different continents (North America, Asia, Africa; Table 1), underscoring the broad international interest toward evidence-based nutrition information. Similar patterns of geolocation-distributions with big share of participants from English-speaking countries but also broad international participation were also observed upon analysis of other hashtags with biomedical significance on X, such as #MedTwitterAI and #PsychTwitter (26, 34).

The performed content analysis of the #NutritionFacts tweets indicate that, in line with our expectations, all among the top 10 most commonly co-occurring hashtags (Table 2) are related to nutrition (#nutrition, #nutritiontips, #healthyfood, #Nutritionist, #HealthyEating, #nutritionplan, and #food) or health (#health, #healthy, and #healthylifestyle). Several of the identified by us co-occurring hashtags (#healthylifestyle, #nutrition, #nutritionist) were also indicated to be among the major hashtags used for social media communications related to nutrition in previous works (29, 30). Similarly, the list of the top trending terms contained words related to nutrition (“eat,” “nutrition,” “food,” “calories”) and health (“make healthy choices,” “experience optimal health”), but even more prevalent was the group of words specifically referring to providing relevant information and labeling (“gives families information,” “labels,” “website,” “consumers deserve updated,” “easy-to-understand nutrition information,” “label”) and general verbs indicating dynamics associated with the shared information (“need,” “unveils,” “enter,” “check,” “learn,” “delay”).

While our initial expectations ware that the shared content would be dominated by nutritionfacts.org articles, the performed analysis of external web domains most frequently hyperlinked in the #NutritionFacts-containing tweets revealed that nutritionfacts.org ranked just on third place with 135 shares. Moreover, none of the most engaging tweet, the most shared image, the most shared video clip, the most shared hyperlink, and the tweet that received most comments, were related to content originating from nutritionfacts.org. Along the same line the account of the nutritionfacts.org founder Dr. Michael Greger was not ranked in the list of the top 10 influencer account of the #NutritionFacts tweets, and most of the top 10 influencer accounts belonged to prestigious organizations such as Mayo Clinic, The United States Food and Drug Administration, and The Academy of Nutrition and Dietetics, among others. Thus, our results indicate that the hashtag #NutritionFacts was well adopted from key healthcare providers for the communication of nutrition-related information, and even was on some cases adopted to increase the visibility of political campaign statements unrelated directly to nutrition (envisaging the most engaging #NutritionFacts tweet in the study period, which was featuring a meme presenting features of Trump supporters in a way resembling “Nutrition facts” on a food label).

While the results obtained from our analysis met our expectation to provide some insights on the posting accounts and the content of the #NutritionFacts tweets, the readers should be aware of the following limitations of this study: (1) X is just one of the most prominent social media networks, but there are also others that are not covered in the current work, such as Facebook, Instagram, Youtube, or TikTok, which likely also contain #NutritionFacts posts; (2) The hashtag #NutritionFacts is just one of the hashtags that can be used for sharing information on X concerning evidence-based nutrition, other examples not analyzed in detail in the current work are #nutritiontips, #HealthyEating, and #healthyfood; (3) Our work does not provide measurable information for acquired new knowledge or behavioral change of the audience of the #NutritionFacts tweets. Thus, to address these limitations and fill-in remaining knowledge gaps, promising avenues for future work can be comparative studies involving several social media platforms, studies comparing the use of several hashtags related to evidence-based nutrition and healthy eating, and research involving measurable parameters of knowledge acquisition or behavioral change of the audience of social media posts containing information on evidence-based nutrition.

## Conclusion

In this work we analyze for the first time the use of the hashtag #NutritionFacts for the promotion of evidence-based nutrition information on X. The archived high visibility (50,348,223 impressions) of the tweets posted during the study period hints on potential impact that #NutritionFacts might have had on the dissemination of science-based nutrition information to the general public. Analysis of healthcare stakeholders that posted the #NutritionFacts tweets reveals that both institutional and individual accounts were broadly participating in the information dissemination, and the user location profiling revealed highly international engagement with countries from three continents, North America, Asia, and Africa, in the top five locations list. The analysis of the top 10 influencer accounts of the #NutritionFacts tweets also revealed both institutional and individual accounts, while the institutional accounts were around twice more prevalent (equaling 7 out of the top 10 accounts). Content analysis of the shared tweets hints on the importance of the provision of relevant and qualitative nutrition-related information to consumers, for the promotion of good health. The present work exemplifies the dissemination potential linked to the use of appropriate hashtags for the promotion of nutrition-related information through X.

## Data availability statement

The original contributions presented in the study are included in the article/supplementary material, further inquiries can be directed to the corresponding author/s.

## Author contributions

MM: Conceptualization, Formal analysis, Investigation, Writing – original draft. TJ: Writing – original draft, Writing – review & editing. MG: Writing – review & editing. FB: Writing – review & editing. AJ: Writing – review & editing. AW: Writing – review & editing. JH: Writing – review & editing. HW: Writing – review & editing. AA: Conceptualization, Formal analysis, Investigation, Project administration, Supervision, Writing – original draft, Writing – review & editing.
